# Stability of a Mutualistic *Escherichia coli* Co‐Culture During Violacein Production Depends on the Kind of Carbon Source

**DOI:** 10.1002/elsc.202400025

**Published:** 2024-09-08

**Authors:** Simon Schick, Tobias Müller, Ralf Takors, Georg A. Sprenger

**Affiliations:** ^1^ Institute of Microbiology University of Stuttgart Stuttgart Germany; ^2^ Institute of Biochemical Engineering University of Stuttgart Stuttgart Germany

**Keywords:** co‐culture, D‐xylose, *E. coli*, mutualism, violacein

## Abstract

The L‐tryptophan–derived purple pigment violacein (VIO) is produced in recombinant bacteria and studied for its versatile applications. Microbial synthetic co‐cultures are gaining more importance as efficient factories for synthesizing high‐value compounds. In this work, a mutualistic and cross‐feeding *Escherichia coli* co‐culture is metabolically engineered to produce VIO. The strains are genetically modified by auxotrophies in the tryptophan (TRP) pathway to enable a metabolic division of labor. Therein, one strain produces anthranilate (ANT) and the other transforms it into TRP and further to VIO. Population dynamics and stability depend on the choice of carbon source, impacting the presence and thus exchange of metabolites as well as overall VIO productivity. Four carbon sources (D‐glucose, glycerol, D‐galactose, and D‐xylose) were compared. D‐Xylose led to co‐cultures which showed stable growth and VIO production, ANT‐TRP exchange, and enhanced VIO production. Best titers were ∼126 mg L^–1^ in shake flasks. The study demonstrates the importance and advantages of a mutualistic approach in VIO synthesis and highlights the carbon source's role in co‐culture stability and productivity. Transferring this knowledge into an up‐scaled bioreactor system has great potential in improving the overall VIO production.

**Abbreviations**
ANT: anthranilate
DVIO: deoxyviolacein
TRP: L‐tryptophan
VIO: violacein

## 1 Introduction

Microbial cells such as *Escherichia coli* offer an environmentally and economically friendly alternative in the synthesis of high‐value chemicals and pharmaceuticals [1, 2]. Several carbon sources from sustainable origins can be utilized by *E. coli*. In addition to glucose, other sugars include the hemicellulose constituents D‐xylose or L‐arabinose, D‐galactose (a moiety of lactose from milk whey), or glycerol (a by‐product from biodiesel production). Among others, *E. coli* is well known for the industrial production of aromatic amino acids [3] or derivatives of aromatic amino acids [4]. For the latter, the introduction of heterologous genes is often necessary.

Violacein (VIO) is a low‐molecular‐weight purple pigment and a natural bisindole product derived from L‐tryptophan biosynthesis [5, 6, 7]. It displays interesting biological properties as it acts as an antimicrobial compound versus many bacteria, protists, or viruses [7, 8, 9]. There is a plethora of further applications of VIO in cosmetics or textile production [10, 11]. VIO (and side products like deoxyviolacein [DVIO]) is naturally produced by Gram‐negative bacteria such as *Chromobacter violaceum* [12, 13] or *Janthinobacterium* sp. [14, 15] which are psychrophilic microorganisms [16, 17]. Both, however, are considered potential pathogens and therefore are not amenable to industrial production of the compounds [7].

**Summary**

- Co‐cultures of two mutualistic *E. coli* mutant strains can be successfully used to produce violacein from various carbon sources.
- Best carbon source is D‐xylose can be derived from sustainable plant biomass.
- Care must be taken to use the optimal starting ratios of both strains to achieve stable bacterial populations and productivity.

Heterologous expression of the *vioABCDE* genes encoding enzymes for the oxygen‐dependent transformation of two tryptophan (TRP) molecules into VIO (or DVIO) has already allowed production with recombinant microbial cells such as yeast [18] or *Corynebacterium glutamicum* [19]. The main recombinant producer, however, is *E. coli* [20, 21, 22, 23, 24, 25, 26, 27, 28, 29, 30].

The predominant approach for VIO production with recombinant *E. coli* cells is the biosynthesis in one producer strain with improved TRP production to provide this as a precursor for VIO [21, 24, 27, 28, 31]. In recent years, the idea of microbial co‐cultures with the metabolic division of labor in the production of chemicals has gained considerable interest [32, 33, 34, 35, 36, 37, 38, 39, 40]. Our group has recently shown that a stable and mutualistic co‐culture of two *E. coli* strains could be obtained [41]. Here, one strain had a genetic block in the *trpD* gene and formed anthranilate (ANT) from glucose. ANT was excreted and served as an auxotrophic compound for the second strain which is unable to synthesize ANT on its own (*trpE* mutation) but can convert ANT to TRP. Excreted TRP then served to complement the growth defect of the first strain. Thus, these strains mutually benefit from each other in an obligatory interdependence [41]. We now went on and studied whether this system may be exploited for the production of the TRP‐based VIO.

Almost all recombinant *E. coli* VIO producers had been grown in monocultures and at temperatures below *E. coli*’s optimal growth temperature of 37°C. This is due to the specific demands of VIO production genes which stem from psychrophilic microorganisms [13, 42]. Recently, by the engineering of the ribosome binding sites, VIO production with *E. coli* from LB media was found to be improved at higher temperatures (30°C and 37°C) [42]; these authors did not report on minimal media with single carbon (C) sources. While the present study was conducted, another group [22] reported on a modular co‐culture for VIO synthesis.

As C sources, several sugars have been reported such as glucose [24], L‐arabinose [21], glycerol [23], and recently D‐galactose (with TRP feeding) [30]. We show below that D‐xylose—which is from sustainable origins—may be preferable over other C sources for a co‐culture production of VIO with *E. coli* strains in a one‐pot shake flask regime. Care should be taken to choose the right ratio of the two partner strains in a co‐culture to avoid an unwanted collapse of the co‐culture, however.

## 2 Materials and Methods

### 2.1 Strain Construction

Strains and plasmids of this study are listed in Table 1. Respective primers are listed in the supporting information (Table S1). The basis for the VIO‐producing co‐culture is the mutualistic *E. coli*—*E. coli* co‐culture (ANT‐3 and TRP‐3) as described previously [41]. Therein, ANT‐3 produces ANT and is auxotrophic for TRP, while strain TRP‐3 is also auxotrophic for TRP but can be supplemented as well with ANT to satisfy its need for TRP. Further modifications were implemented via a plasmid‐based CRISPR/Cas9 method as described earlier [43]. The deletion of chromosomal genes for L‐arabinose degradation (Δ*araBAD*::FRT) was created by the lambda‐red‐based recombineering method [44]. Flippase recognition target (FRT)‐flanked kanamycin cassettes were removed by the transformation of pCP20 [45]. ANT‐5 was created by deleting the *lacZ* and *araBAD* genes. The partner strain TRP‐5 received a chromosomally integrated *vioD* gene (from *C. violaceum* ATCC 12472, codon‐optimized for *E. coli* K‐12) replacing parts of the *fuc* locus, in addition to the *araBAD* deletion. For VIO production, the TRP‐5 strain was transformed with pVioABCE‐km [21], comprising the *vioABCE* genes from *C. violaceum*, while ANT‐5 was transformed with pVio_empty_‐km to allow the use of kanamycin in the medium. Genetic changes were verified by PCR (gene deletions) and DNA sequencing (gene insertions). All strains were stored in glycerol stocks at −70°C.

**TABLE 1:** List of strains and plasmids.

Name	Relevant genotype	Source
Strains		
*E. coli* K‐12 LJ110	W3110 *fnr^+^*	[46]
ANT‐3	LJ110 Δ*trpD* Δ*tnaA::FRT* Δ*trpR::FRT‐Km^R^‐FRT*	[41]
ANT‐3a	LJ110 Δ*trpD* Δ*tnaA::FRT* Δ*trpR::FRT*	This study
ANT‐4	ANT‐3a Δ*lacZ*	This study
ANT‐5	ANT‐4 Δ*araBAD::FRT*	This study
TRP‐3	LJ110 Δ*trpE* Δ*trpR::FRT* Δ*tnaA::FRT*	[41]
TRP‐4	TRP‐3 Δ*fuc::P_tac_‐vioD*	This study
TRP‐5	TRP‐4 Δ*araBAD*	This study
Plasmids		
pCas	*repA101(ts) km P_cas_‐cas9 P_araB_‐Red lacI^q^*	[43]
pTarget‐vioD	*cat sgRNA‐*Δ*fuc::P_tac_‐vioD*	This study
pTarget‐araBAD	*cat sgRNA‐*Δ*araBAD*	This study
pKD46	*P_BAD_gam‐bet‐exo*	[44]
pCP20	*flp*	[45]
pCO1	*Km^R^*	[47]
pVioABCE‐km	*P_BAD_‐vioABCE Km^R^*	[21]
pVio_empty_‐km	*Km^R^*	This study

### 2.2 Cultivation and Media Composition

#### 2.2.1 Culture Medium

All strains were cultivated in minimal medium (MM) [48] with 20 mg L^−1^ thiamin‐hydrochloride and 5 g L^−1^ of carbon source (either glucose, glycerol, galactose, or xylose), adjusted to pH 7.0 with 1 mol L^−1^ HCl. Kanamycin was added to a final concentration of 0.05 g L^−1^ to prevent plasmid loss. For VIO production, 20 mmol L^−1^ L‐arabinose and 0.1 mmol L^−1^ IPTG were supplemented as inducers of the *vio* genes at the beginning of the main cultivation.

#### 2.2.2 Shake Flask Cultivation

Pre‐culture flasks (20 mL MM in a 250 mL shake flask with a cotton plug) were inoculated from a single colony of either ANT‐5 + pVio_empty_‐km or TRP‐5 + pVioABCE‐km from a MM agar plate. As these strains are auxotrophic, 0.1 g L^−1^ ANT (for TRP‐5) or TRP (for ANT‐5) were supplemented to the medium. Incubation took place at 30°C and 200 rpm for 18–42 h to reach an optical density of ∼4 at 600 nm (OD_600_). For the main co‐culture in flasks (50 mL MM in a 500 mL shake flask with a cotton plug), cell suspensions of both pre‐cultures were washed with 500 µL MM to remove residual ANT or TRP and inoculated with a combined OD_600_ of 0.2. The initial inoculation ratio (ANT‐5/TRP‐5) was determined as shown: 50/50 (OD_600_ of 0.1/0.1), 70/30 (OD_600_ of 0.14/0.06), and 30/70 (OD_600_ of 0.06/0.14). Incubation took place at 30°C or 25°C (for optimal VIO production) and 200 rpm for up to 120 h in a shaking incubator until the carbon source was depleted. If not indicated otherwise, all co‐cultures were performed in triplicates.

### 2.3 Strain Ratio Identification

To monitor the strain ratio of ANT‐5 and TRP‐5 within the cultivation flask, cell suspensions were plated out on MacConkey agar plates with 10 g L^−1^ lactose and incubated at 37°C for 24 h. The *lacZ* deletion in ANT‐5 leads to pale colonies as the strain cannot degrade the lactose, while TRP‐5 forms deep red colonies (see Figure S1). The ratio was determined by counting 100–200 pale and red colonies on each plate (minimum: 50 colonies counted).

### 2.4 Violacein Extraction

For the extraction of VIO, method adapted from Rodrigues et al. [23], the cell pellet was mixed with 1 mL of pure ethanol and disrupted with glass beads under eight‐shaped shaking movements (Silamat S6, Ivoclar: 4500 rpm, 30 s). The glass beads and cell debris were removed by centrifugation (14,000 rpm, 1 min) and the supernatant (VIO extract) was collected for analytics. This step was repeated until no violet color was visible in the supernatant. The supernatant of the cell culture (ANT, TRP, and carbon sources) or the supernatant of extracts (VIO) were used for all HPLC analysis.

### 2.5 Analytical Methods

#### 2.5.1 Determination of Optical Density (OD_600_) and Cell Dry Weight

The growth of the strains was monitored by photometric measurement (Cary 60 UV‐Vis, Agilent) at OD_600_ during cultivation. The cell dry weight (CDW) was determined by a correlation factor between OD_600_ and CDW, as calculated in previous work [41].

#### 2.5.2 Violacein Determination by HPLC

VIO analytics were performed by an HPLC method described earlier [20]. Therefore, an UHPLC (Dionex UltiMate 3000, Thermo Fisher Scientific) with a C‐18 column (Luna C18(2), 5 µm, 250 × 4.6 mm, Phenomenex) was used. The temperature of the column oven was at 30°C and the samples were cooled to 5°C in the sampler unit. The injection volume of the VIO sample was 10 µL. An isocratic flow of 50% (v/v) ethanol (mobile phase) with a flow rate of 0.5 mL min^−1^ over 20 min was used. A diode array detector (DAD‐3000) was used at a wavelength of 258 nm, one of the absorption maxima of VIO [5]. As external standards commercial VIO (Cayman Chemical Company) and DVIO (Santa Cruz Biotechnology) were used, dissolved in pure ethanol. Note that the quantification of VIO in this work is displayed by the combined concentrations of VIO and DVIO.

#### 2.5.3 Anthranilate and Tryptophan HPLC

The quantification of ANT and TRP concentrations was performed similarly by an HPLC method (gradient see Figure S2) used in a previous publication [41].

#### 2.5.4 Sugar HPLC

The consumption of carbon sources during the cultivation was measured via sugar HPLC method adapted from Nargesi et al. [49]. The HPLC (Infinity 1260, Agilent Technologies) with an organic acid column (Organic Acid‐Resin, 300 × 8 mm, Chromatographie‐Service GmbH) was used. The temperature of the column oven was at 40°C, the sampler unit was cooled to 10°C. The injection volume of the sugar samples was 5 µL. An isocratic flow of 5 mmol L^−1^ sulfuric acid (mobile phase) with a flow rate of 0.8 mL min^−1^ over 25 min was used. A refractive index detector (RID 1260) was used for the sugar detection. External commercial standards were used for calibration.

### 2.6 Calculation of Rates and Yields

The mean biomass‐specific production rates of compound i $q_{i}$ [mg (g h)^−1^] or [mg (OD_600_ h)^−1^] was calculated based on the concentration difference of the produced compound $\Delta c_{i}$[mg L^−1^] between two measurements, related to the mean biomass in that interval (optical density $\bar{c_{X}}$  [OD_600_] or concentration $\bar{c_{X}}$ [g L^−1^]; correlation factor as described recently [41]) and the time difference Δt [h].

$$q_{i}=\frac{\Delta c_{i}}{\bar{c_{X}}\cdot \Delta t}$$

For the calculation of the specific VIO production rate, HPLC‐quantified VIO concentrations were used. The VIO production was related to the total co‐culture population (ANT‐5 and TRP‐5). The L‐tryptophan supply rate of strain TRP‐5 for ANT‐5 was calculated based on the estimated requirement of this amino acid for ANT‐5 to realize the growth that was experimentally determined (assumption: 72% protein CDW^−1^ [g g^−1^] [50] and an L‐tryptophan protein fraction of 1.1% [51]). The estimated L‐tryptophan production was related to the mean biomass concentration of the producer strain TRP‐5. The VIO yield from substrate $Y_{\mathrm{VIO}/S}$ [mg g^−1^] in a considered time period was calculated based on the concentration difference of produced VIO $\Delta c_{\mathrm{VIO}}$[mg L^−1^] related to the concentration difference of the carbon source used $\Delta c_{S}$[g L^−1^] in that interval.

$$Y_{\mathrm{VIO}/S}=\frac{\Delta c_{\mathrm{VIO}}}{\Delta c_{S}}$$

## 3 Results

### 3.1 Considerations for Strain Construction and Implementation

**FIGURE 1:**
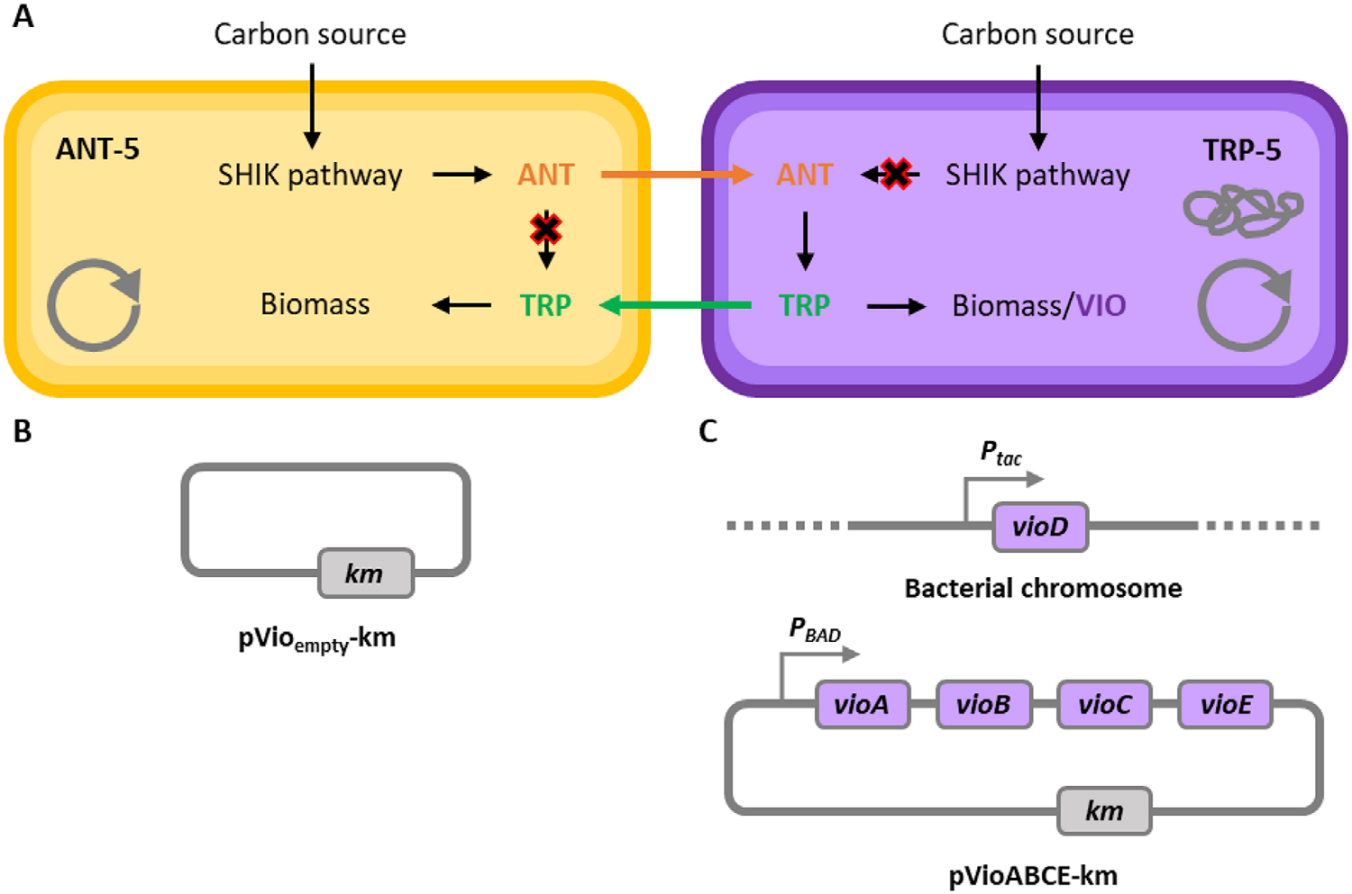
Basic principle of the violacein‐producing, synthetic mutualistic co‐culture. (A) Simplified principle of the interaction of the strains ANT‐5 (ANT‐supplier) and TRP‐5 (TRP‐supplier, VIO‐producer), based on the previously described co‐culture (ANT‐3/TRP‐3) [41]. (B) Empty plasmid backbone of pVioABCE‐km, providing kanamycin resistance for ANT‐5. (C) Integration of the vioD gene (from *Chromobacterium violaceum*) into the bacterial genome (fuc locus) of TRP‐5, plus transformation of pVioABCE‐km [21] for complete VIO production. Both strains were additionally modified by deletion of their *araBAD* genes to not use L‐arabinose (inducer of PBAD) as a carbon source.

We adopted our successful approach of using two *E. coli* strains, one with a genetic block (a partial deletion in the *trpD* gene) that leads to excretion of ANT, and the other which is ANT‐auxotrophic but can produce and excrete TRP (deletion of *trpE* gene). The strains were genetically further developed by introducing deletions of the *trpR* (for the TRP repressor) and *tnaA* (for tryptophanase) genes to avoid undesired effects of repression by TRP or degradation of TRP (see Table 1) [41]. For VIO production, the TRP producer strain was chromosomally equipped with *vioD* gene (under P_tac_ control) and with a plasmid carrying *vioABCE* genes from *C. violaceum* [21]. To allow a co‐culture in the presence of the antibiotic kanamycin (selection marker for the pVioABCE‐km plasmid), the ANT producer was transformed with the empty vector (pVio_empty_‐km) (see Figure 1). To distinguish the two strains in a mixed culture and to be able to determine the ratio of the two strains in a co‐culture, the ANT producer was made lactose‐negative (Δ*lacZ*). The relative abundance of the two strains in co‐culture could thus be assessed by taking samples from the different growth phases and plating them out (after appropriate dilution) on MacConkey agar plates with lactose (1%). The ANT producers developed pale colonies, whereas the Lac+ TRP producers formed red colonies (see Figure S1).

As the *vioABCE* genes are under the control of a P*_araBAD_* promoter, L‐arabinose was added to the culture media for induction; to avoid the further catabolism of this pentose, the strains ANT‐5 and TRP‐5 carry deletions of the *araBAD* genes (see Table 1). Further, IPTG (0.1 mM final concentration) was used to induce the chromosomally encoded *vioD* gene. As expected, the cell suspension of strain TRP‐5 when equipped with the pVioABCE‐km plasmid developed the typical deep‐violet color indicative for the successful expression of the *vio* genes (see Figure S3). This, however, was only the case when TRP‐5 grew (in the presence of L‐arabinose as inducer) on LB agar plates, or on minimal agar plates with a single C source and supplementation of an inducer and either ANT or TRP.

### 3.2 Co‐Cultures of Strains Allow Production of VIO From Various Carbon Sources

We then tested whether a stable co‐culture of the two strains ANT‐5 and TRP‐5 with their respective plasmids could be established. Therefore, both strains were first grown separately (as overnight cultures) and were then mixed in a 1:1 (50:50) ratio (based upon the OD_600_ values determined from the precultures). Incubation took place in shake flasks at 25°C in MM with 5 g L^−1^ glucose. Without addition of inducers, the co‐culture behaved as stable over the time course of 24 h (see Figure S4). This verified that these two strains, indeed, fed each other, when not induced for VIO production, similar to what we have reported before [41].

**FIGURE 2:**
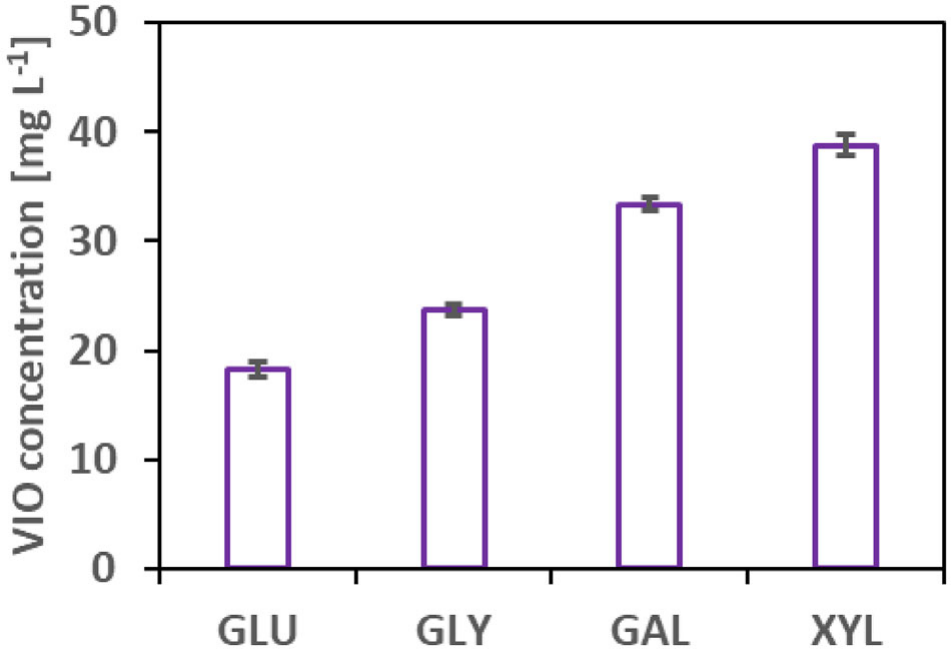
Final violacein concentration of shake flask experiments in minimal medium with novel co‐culture (ANT‐5/TRP‐5; initial ratio: 50/50) at 30°C with four different carbon sources (glucose, glycerol, galactose, and xylose). The data presented are from three biological replicates (two replicates for glucose).

A recent study showed that synthetic *E. coli* consortia were able to synthesize up to roughly 50 mg L^−1^ of VIO in shake flask cultivation with 10 g L^−1^ D‐galactose at 30°C after optimizing the process (inoculation ratios, surfactant addition, and induction time point) [22]. To get a first understanding of the general VIO production ability and co‐culture dynamics of our mutualistic co‐cultures, the consortium was grown on four different C sources (D‐glucose, glycerol, D‐galactose, and D‐xylose; 5 g L^−1^ intended starting concentration each), induced throughout with L‐arabinose and IPTG. After cultivation, the final VIO concentrations were ∼18 mg L^−1^ for D‐glucose and ∼24 mg L^−1^ with glycerol, the best values were achieved with D‐galactose and D‐xylose, ∼33 and 39 mg L^−1^, respectively (see Figure 2).

**FIGURE 3:**
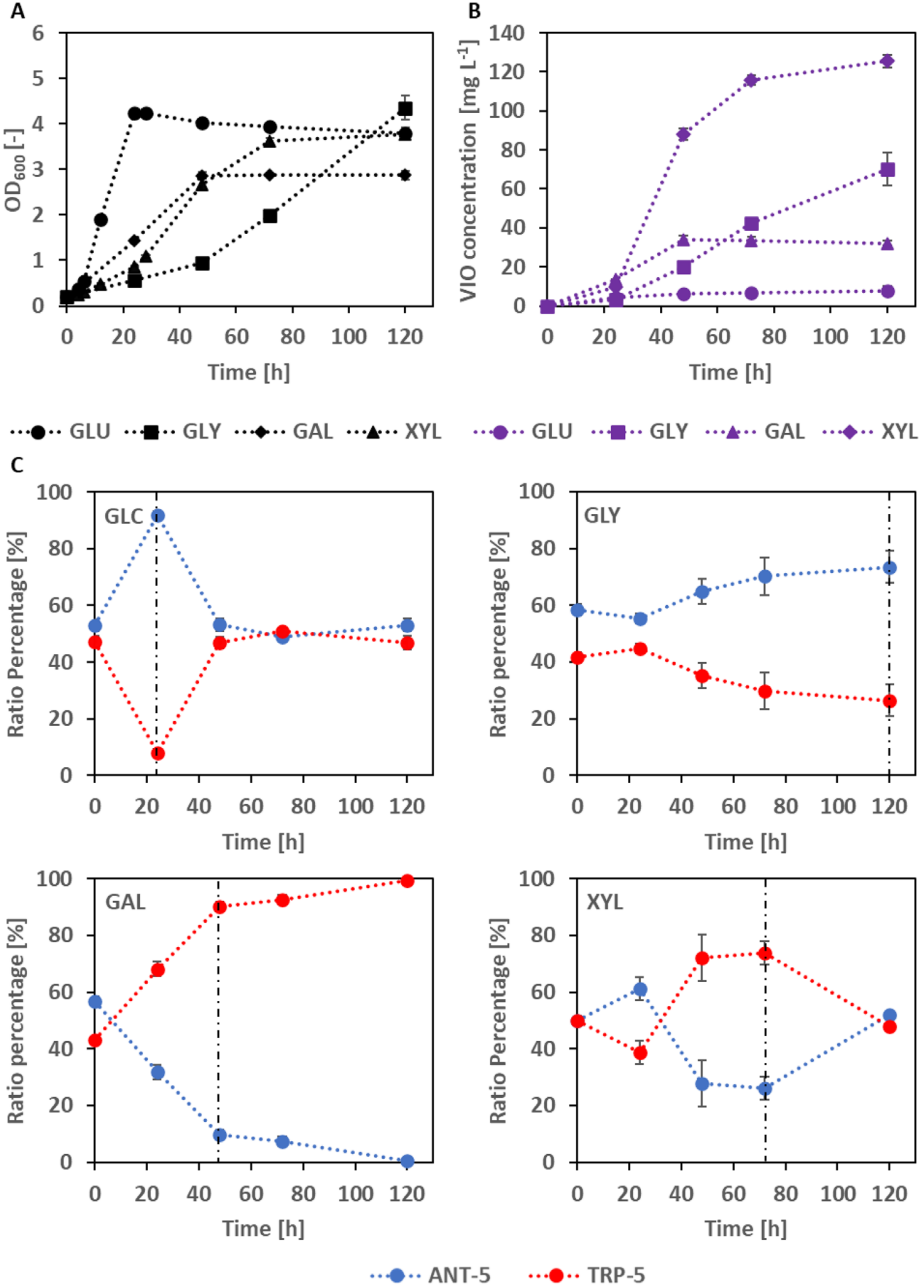
Shake flask experiment in minimal medium with novel co‐culture (ANT‐5/TRP‐5; initial ratio: 50/50) for violacein production at 25°C with four different carbon sources (glucose, glycerol, galactose, and xylose). (A) Optical density at 600 nm, (B) violacein concentration over time, and (C) strain ratio percentages over cultivation time (determined on MacConkey agar plates). The vertical dashed line represents the end of the carbon source. The data presented are from three biological replicates.

We next compared the growth, VIO production, and population structure over a period of up to 5 days (120 h) in shake flasks to further exploit the shown potential of these mutualistic co‐cultures. As VIO producers are known to be temperature‐dependent, we chose 25°C for further experiments. The four different C sources were compared, again. As expected, growth on D‐glucose was fastest and the cell cultures reached an OD_600_ of ∼4.2 after already 24 h of incubation (Figure 3A). Growth on the three other C sources lagged clearly behind, glycerol as C source allowing the slowest growth. Final OD_600_ values ranged between ∼2.9 (D‐galactose), ∼3.8 (D‐glucose, D‐xylose), and ∼4.4 (glycerol). Carbon sources were depleted in quite different fashions. Glucose was consumed after 24 h, galactose after 48 h, xylose after 72 h, and glycerol at the end of incubation (120 h) (see Figure 3C). VIO concentrations were low in the D‐glucose shake flasks (lower than 8 mg L^−1^ after 120 h) (Figure 3B). D‐Galactose yielded more VIO with a maximum of 34 mg L^1^ reached already after 48 h. Glycerol showed VIO production which was correlated to cell growth and up to 70 mg L^−1^ after 120 h. Finally, D‐xylose gave the best results. VIO accumulated after a lag of ∼24 h but then developed continuously, reaching a maximal value of ∼126 mg L^−1^ after 120 h.

### 3.3 Population Structures Vary Enormously Depending on C Source

As described above, we determined the percentage of the two strains ANT‐5 and TRP‐5 in the total population in the shake flask cultivations by plating out samples. To discern between strains, MacConkey agar plates with added lactose were used. We intended to establish 50:50 ratios at the starting time by mixing equivalent cell numbers as determined from the overnight cell densities. It should be noted that slight deviations from the intended 50:50 start‐ratios were observed (see Figure 3C, 0 h data points). During growth on glucose, strain ANT‐5 reached a share of almost 90% after 24 h. At that point of time no residual glucose was detectable and the co‐culture had reached its maximum OD_600_ value. Thereafter, ANT‐5's share went down to about 50% and stayed thus till the end of incubation after 120 h. Growth on glycerol started with an actual ratio of roughly 58% (ANT‐5):42% (TRP‐5). ANT‐5 stayed at this share after 24 h and eventually reached about 74% at the end of incubation. TRP‐5 went from 42% in the beginning to 26% at the end of incubation. D‐Galactose as C‐source displayed a reverse population structure development. TRP‐5 started at about 43% and went up to 90% after 48 h. At this point of time, galactose was consumed and the maximum OD value was noted. Afterward, the percentage of TRP‐5 still went higher (up to >99%) at the end of incubation.

D‐Xylose retained a more stable ratio. Both strains had roughly a 50:50 share in the beginning. Thereafter, ANT‐5 went up to about 61% after 24 h and then dropped to values below 30% at 72 h, which was the time point when no more D‐xylose was present and the OD value had reached its maximum. TRP‐5 instead went from 50% via 39% to 74% at 72 h. Eventually, both strains had an almost equal share at the end of incubation. It should be noted here that three biological replicates were run which all gave very similar trajectories.

### 3.4 Variations in the Initial Ratios Result in Quite Different Trajectories in Population Structure

**FIGURE 4:**
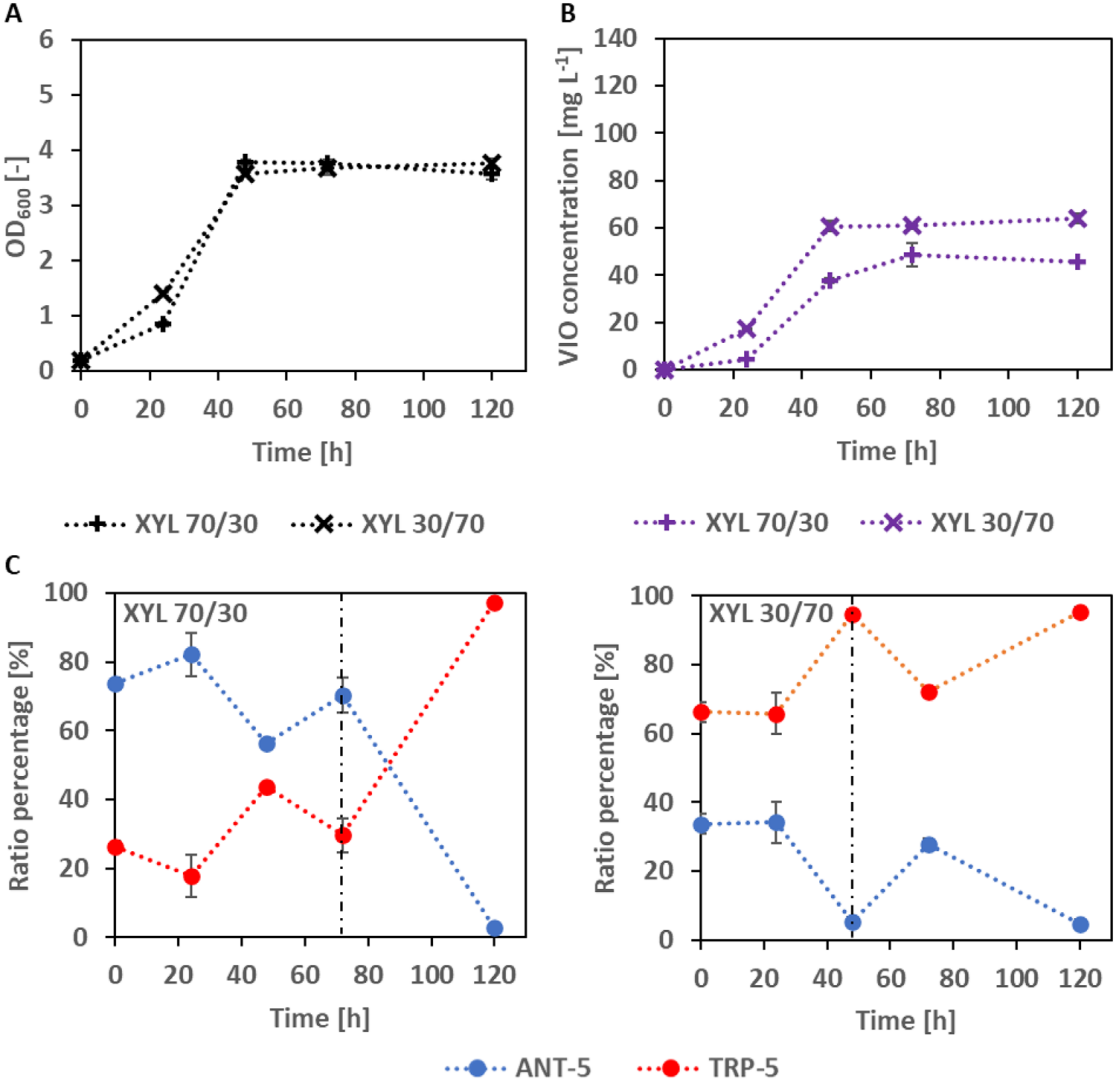
Shake flask experiment in minimal medium with xylose with novel co‐culture (ANT‐5/TRP‐5; initial ratio: 70/30 and 30/70) for violacein production at 25°C. (A) Optical density at 600 nm, (B) violacein concentration over time, and (C) strain ratio percentages over cultivation time (determined on MacConkey agar plates). The vertical dashed line represents the end of the carbon source.

As D‐xylose had turned out as most favorable C source for a stable co‐culture, we wanted to know what the influence of variable start ratios was. We therefore ran shake flask incubation with changed initial (calculated) ratios of 70% (ANT‐5):30% (TRP‐5), or to the reverse (30% [ANT‐5]:70% [TRP‐5]). As can be seen, the two mixing ratios resulted in very similar growth trajectories (Figure 4A) with final OD values of about 4; the C source was consumed after 72 h.

The VIO product yields, however, were different with the 30/70 ratio as indicated by higher VIO values throughout the incubations over 120 h. The absolute VIO values, however, were less (∼64 and ∼46 mg L^−1^, respectively) than in the 50/50 ratio experiments. The measured strain ratios showed quite different behavior. In the 70/30 experiments, ANT‐5 increased slightly at 24 h to 82%, then dropped to values of 56% and 70% after 48 and 72 h. Finally, the share of ANT‐5 was less than 3% at the end of incubation. TRP‐5 instead went from 26% to <20%, then increased to 44% and to 30% at 72 h. Then, curiously, strain TRP‐5 completely overtook the population to have a share of >97% at the end of incubation. The 30/70 experiment on the other side showed an unbalanced ratio after C source consumption (after 48 h), but after 120 h a similar ratio than the other approach was detected (see Figure 4C).

**FIGURE 5:**
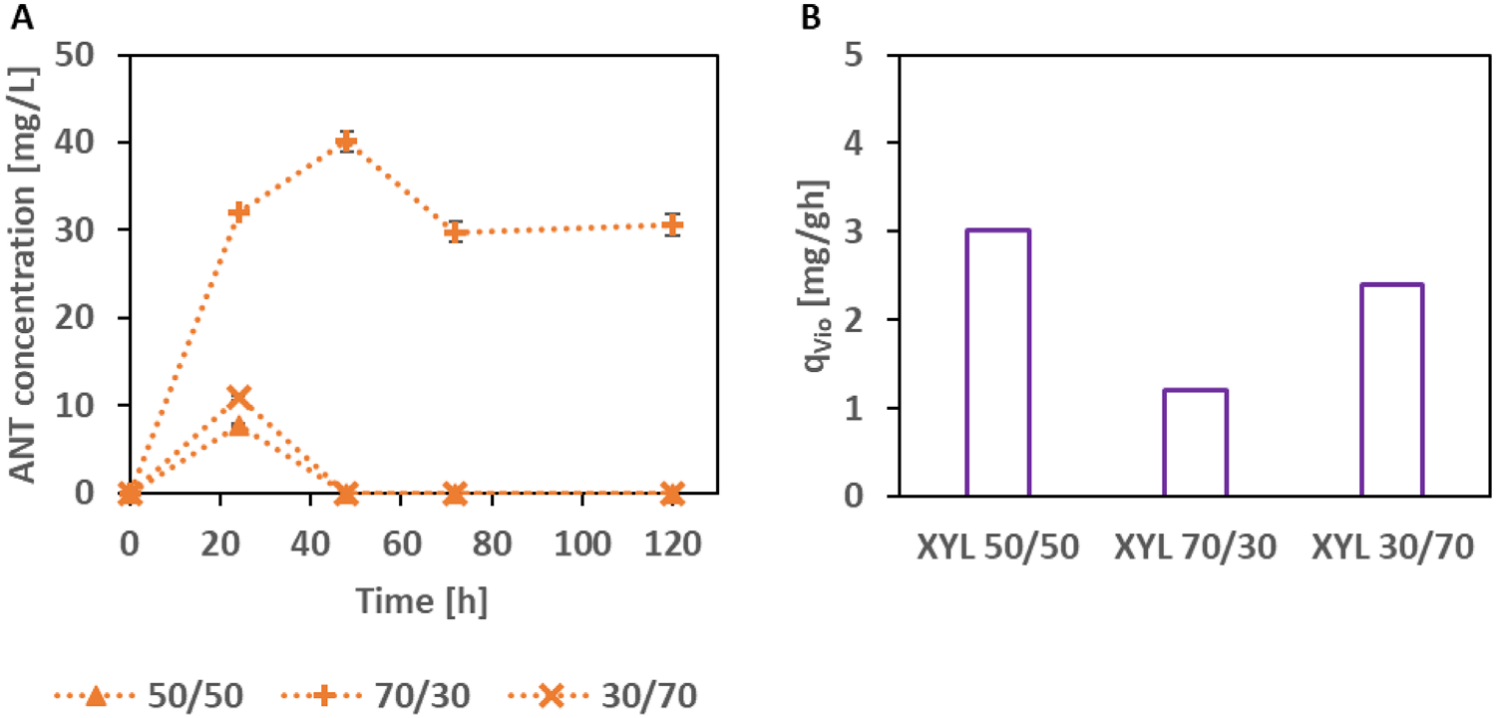
Analytics of the xylose at different initial ratios (50/50, 70/30, and 30/70). (A) Anthranilate concentrations during cultivation time. (B) Biomass‐specific violacein production rate (q_VIO_) calculated from mean values.

We analyzed the concentrations of ANT and TRP in the supernatants of the three xylose co‐cultures. Free TRP could not be detected at any time (detection limit: 0.1 mg L^−1^). ANT concentrations peaked at 24 h with less 12 mg L^−1^ with the 50:50 and 30:70 ratio co‐cultures (Figure 5A). The 70:30 co‐culture showed ANT concentrations above 30 mg L^−1^ at 24, 48, 72, and 120 h. We also determined the biomass‐specific VIO production rate (q_VIO_). As can be seen from Figure 5B, the 50:50 initial ratio gave highest q_VIO_ of about 3.0 and 2.4 mg g^−1^ h^−1^ for the 30:70 ratio. The 70:30 ratio gave slightly more than 1.2 mg g^−1^ h^−1^.

## 4 Discussion

In a pioneering work, two recombinant *E. coli* strains carrying different plasmids (pVioABX, whereby X contained the *vioE* gene, and pVioCD, respectively) were co‐cultured in 2xYT medium and shown to produce a mixture of compounds consisting of VIO, DVIO, and proviolacein [52]. This approach, however, was not a classical co‐culture but rather served to enlighten the biosynthetic pathway of VIO.

A group recently used a commensalistic co‐culture to produce VIO [22]. They identified several optimizing parameters regarding the carbon source used, the inoculation strain ratio, the time of induction and the addition of surfactants. A final VIO concentration of ∼50 mg L^−1^ was achieved and a final overall OD_600_ of about 9 (already after 24 h, presumably due to depletion of the initial 10 g L^−1^ supplied C source). In comparison, using the same carbon source (galactose, set starting concentration 5 g L^−1^) and temperature, our mutualistic co‐culture achieved substantially higher VIO production performance parameters both in terms of substrate‐to‐product yield and biomass‐specific VIO productivity. Since the respective reference values are not given for the commensalistic approach [22], we calculated the values based on the data isolated from the corresponding figure (Figure 4D in [22]). Without pursuing any optimization strategies, our synthetic mutualistic co‐culture achieved a ∼48% higher yield value Y_VIO/GAL_ (mg g^−1^) and a mean specific production rate q_VIO_ (mg [OD_600_ h]^−1^) of ∼2.2 × the commensalistic reference, suggesting that our approach is promising. It is worth mentioning that comparing the performance parameters after the 24 h mark (end of growth in [22]) would lead to an even more evident benefit.

**FIGURE 6:**
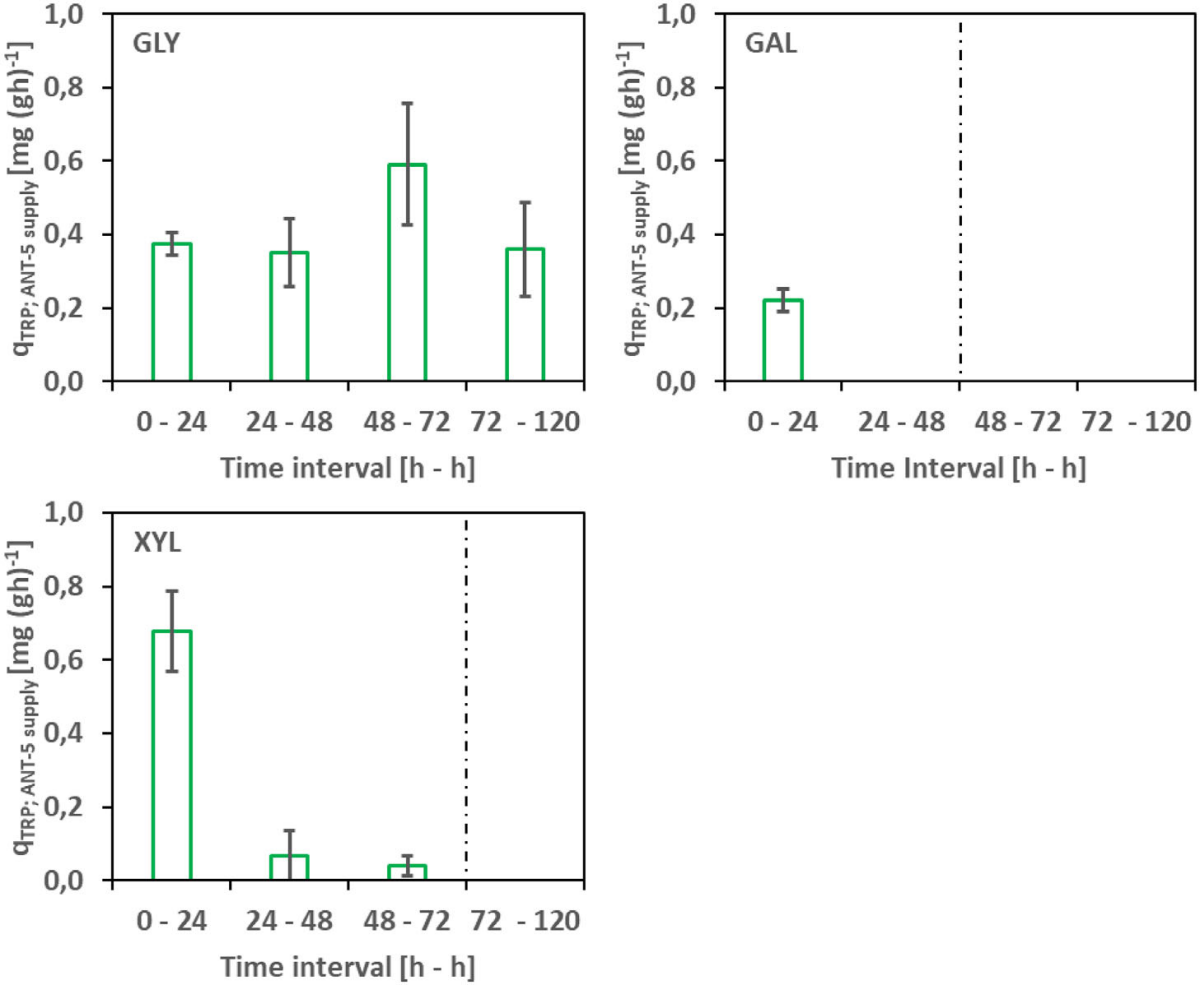
Estimated specific L‐tryptophan providing rate to strain TRP‐5 for the growth of the auxotrophic strain ANT‐5 (q_TRP; TRP‐5 for ANT‐5_) in a common shaking flask environment using different carbon sources. The rates are calculated based on the results presented in Figure 3 (for details, see Section 2). The dashed lines represent the point after carbon source depletion, which was therefore not considered. Error bars represent the standard deviation of biological triplicates.

As a VIO production improvement strategy, we examined the well‐known approach of temperature reduction. When lowering the temperature to 25°C, the glycerol and D‐xylose batch cultivations even reached final values of ∼70 and ∼126 mg L^−1^ of VIO. In the mentioned *E. coli* co‐culture, D‐galactose was the most favorable C source [22], while our mutualistic consortium achieved a 3.9‐fold higher final VIO concentration when supplied with D‐xylose compared to D‐galactose. Our findings showed that VIO production is clearly determined by the cultivation temperature (for glycerol and D‐xylose), as shown by several studies before [12, 42, 53]. The stable growth of the mutualistic co‐culture depends on the cross‐exchange of metabolites. We found that the different carbon sources permit this process to occur to different degrees, resulting in different population dynamics. The ANT producer ANT‐5 consistently provides an oversupply of ANT in almost all cases (except D‐xylose 48 h time point), which can be deduced from the different levels of ANT accumulation in the medium (data not shown). For the TRP auxotrophic strain ANT‐5, no such TRP oversupply was detected. However, in the case of GLY as carbon source, a sufficient supply of TRP seems to be ensured, as indicated by relatively stable population ratios. In contrast, the decrease of ANT‐5 in the total population of the GAL and XYL approaches (from 24 h) points toward an insufficient TRP supply (see Figure 6).

Comparing the assumed TRP supply rates of TRP‐5 to ANT‐5 with those of the stable GLY approach (see Figure 6), it was found that the TRP supply rates are permanently or temporarily below the value of q_TRP; TRP‐5 for ANT‐5_ of ∼0.42, which is considered as necessary for stable population dynamics (mean value GLY approach).

The strain ratio analysis revealed that a stable co‐culture resulted in a higher production of VIO, which was concurrently dependent on the kind of C source. During cultivation especially the use of D‐xylose maintained stability that led to higher VIO concentrations. This underlines the complexity of different co‐cultures, but nonetheless shows the potential of our mutualistic approach. While other groups surpassed our VIO production with values up to 4.45 g L^−1^ from monocultures grown on several C sources like D‐glucose and glycerol in bioreactors [23, 26], we could expand feasible operating strategies by moving from shake flasks to spatially separated, interconnected two‐compartment bioreactor systems with strain‐specific temperature and process conditions [41, 53].

Inoculation ratios of co‐cultures also have an impact on the productivity and stability of co‐cultures [22, 41]. Shifting the initial ratio of D‐xylose grown co‐culture strains ANT‐5 and TRP‐5 to 70/30 and 30/70 gave new insights. The biomass‐specific VIO production rate (q_VIO_) was generally lower than the 50/50 co‐culture, especially the 70/30 approach. However, stability of the co‐culture was not the reason for the lower production rate, as it had a nearly 50/50 ratio at the complete consumption of the C source. An ANT accumulation was detectable in the extracellular metabolite concentration, which was not fully consumed by the TRP‐5 VIO producer strain, compared to the others. The C source here was mainly used for ANT production than for VIO production.

Our results indicate that the kind of carbon source and co‐culture stability are closely related and are crucial parameters for VIO productivity, and for the state of metabolic cross‐feeding balance. The impact of metabolic cross‐feeding at the core of the mutualistic co‐cultures was already highlighted in our previous work [41]. This mutualistic and stable VIO‐producing *E. coli* co‐culture on D‐xylose was able to produce a final concentration of ∼126 mg L^−1^ of VIO in a shake flask batch approach. In the future, the co‐culture with its promising characteristics could be applied to a scaled‐up continuous bioreactor fermentation for VIO production. Additionally, the co‐culture principle is not limited to VIO production only but has the potential to also synthesize other valuable molecules.

## Conflicts of Interest

The authors declare no conflicts of interest.

## Supporting information



Supporting Information

## Data Availability

The data that support the findings of this study are available from the corresponding author upon reasonable request.
