# Supplementary material for: Stability of a Mutualistic Escherichia coli Co‐Culture During Violacein Production Depends on the Kind of Carbon Source
Source: Eng Life Sci. 2024 Sep 8;24(10):e202400025. doi: 10.1002/elsc.202400025 (PMC11464148; doi:10.1002/elsc.202400025)
Supplement: Supplementary file 1 — Supporting Information [file ELSC-24-e202400025-s001.docx]

**Supporting Information**


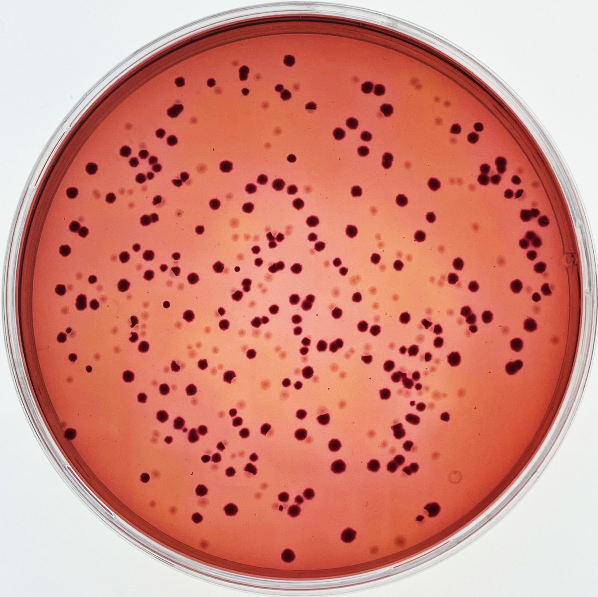


Figure S1: Strain ratio detection via MacConkey with 10 g L^-1^ lactose agar plate after incubation. ANT-5 = pale colonies, TRP-5 = deep red colonies. (+ 40 % contrast)


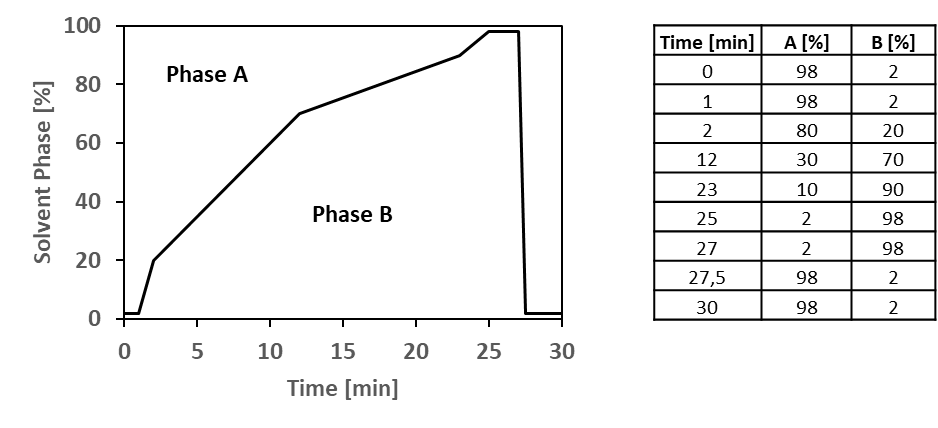


Figure S2: Gradient curve of Phase A (0.1 % trifluoroacetic acid in H_2_O) and Phase B (0.1 % trifluoroacetic acid in methanol) for anthranilate and L-tryptophan HPLC.


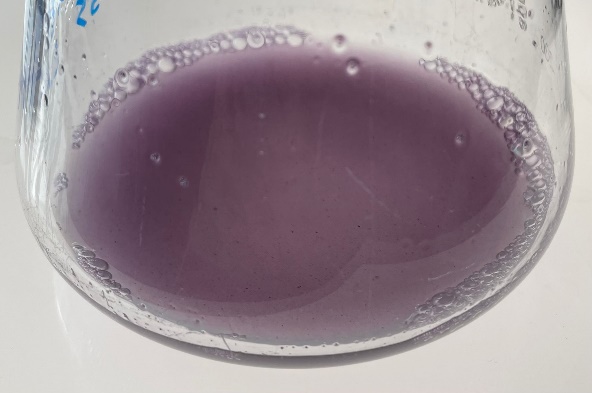


Figure S3: Shake flask with VIO-producing and supplemented TRP-5 with pVioABCE-km in minimal medium at 25 °C after 48 h of incubation.

**
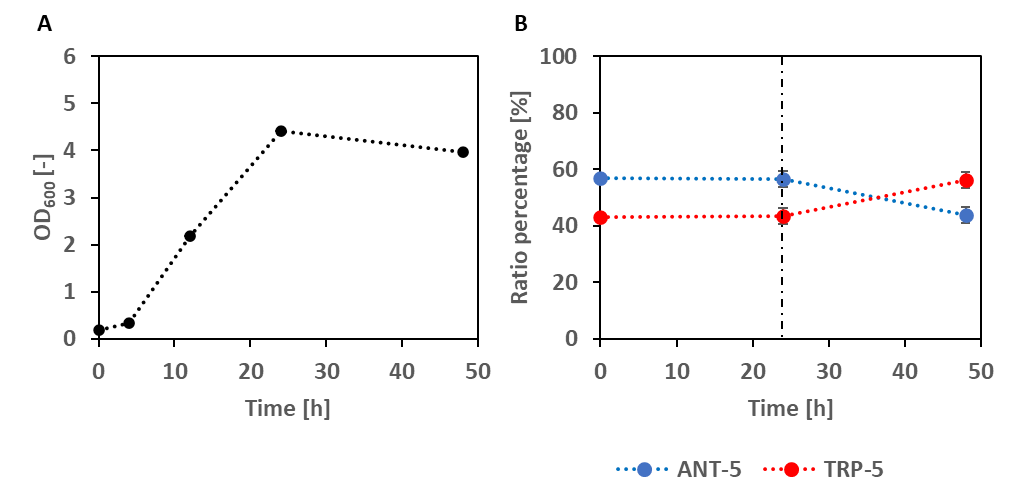
**

Figure S4: Co-culture growth on minimal medium with 5 g L^-1^ glucose at 25 °C, uninduced (in biological duplicates). (A) Optical density at 600 nm (C) and strain ratio percentages over cultivation time (determined on MacConkey agar plates). The vertical dashed line represents the end of carbon source.

Table S1: List of primers used in this study.

| Name | Sequence 5‘-3‘ | PCR template | PCR product |
| --- | --- | --- | --- |
| LacZ-F1 | TTTTGAATTCTTCACACAGGAAACAGCTATGACC | *E. coli* K-12 LJ110 | 5‘-*lacZ*‘-BamHI |
| LacZ-R1 | TTTTGGATCCCGGATTGACCGTAATGGGATAG |  |  |
| LacZ-F2 | TTTTGGATCCGGAAAACCTACCGGATTGATG | *E. coli* K-12 LJ110 | 3‘-*lacZ*‘-BamHI |
| LacZ-R2 | TTTTAAGCTTGCTCCAGGAGTCGTCGCCACCAATC |  |  |
| LacZ-F1 | TTTTGAATTCTTCACACAGGAAACAGCTATGACC | 5‘-*lacZ*‘+3‘-*lacZ*‘ (lig.) | Δ*lacZ* fragment |
| LacZ-R2 | TTTTAAGCTTGCTCCAGGAGTCGTCGCCACCAATC |  |  |
| AraBAD-FRT-F | CGTCACACTTTGCTATGCCATAGCATTTTTATCCATAAGATTAGCGGATCCGTTGTGTAGGCTGGAGCTGCTTCG | pCO1 | 5‘-araBAD’-FRT-kmR-FRT-araBAD’-3’ |
| AraBAD-FRT-R | GTATAGCCTGGTTTCGTTTGATTGGCTGTGGTTTTATACAGTCATTACTGCCATATGAATATCCTCCTTAGTTC |  |  |
| VioD-fuc-F | TGCTGTGCTCACTGTTTTTTCTTTGGGCGGTAGCCAATAACCTTAACGACATTTTATTACCGACATCATAACGGTTCTGGC | pVioD-cm | Δ*fuc::vioD*  fragment |
| VioD-fuc-R | CAGCATGGAGGCGAGAGTGATAAAGTCTGCGCCAACGTGGCCGATGGTCAGAACCCCCCTGCAGGTCGACTCTAGAGGATC |  |  |
| AraBAD-F | AACCTTTCATTCCCAGCGGTCGGTCG | *E. coli* K-12 LJ110 | 5‘-*araBAD*‘-linker |
| AraBAD-L1 | AGGAGACTTTCTGATGGCGGTGAAGCGTCAGGTAGGATCCGCTAATCTTATG |  |  |
| AraBAD-L2 | CACCGCCATCAGAAAGTCTCCTTACGGGCAGTAATGACTGTATAAAACC | *E. coli* K-12 LJ110 | 3‘-*araBAD*‘-linker |
| AraBAD-R | AGTGCCATTGTGCATATCACCCTCGAC |  |  |
| AraBAD-F | AACCTTTCATTCCCAGCGGTCGGTCG | 5‘ + 3‘-*araBAD*‘ linker | Δ*araBAD* fragment |
| AraBAD-R | AGTGCCATTGTGCATATCACCCTCGAC |  |  |
